# Accuracy of Imputation for Apolipoprotein E ε Alleles in Genome-Wide Genotyping Data

**DOI:** 10.1001/jamanetworkopen.2019.19960

**Published:** 2020-01-24

**Authors:** Eero Vuoksimaa, Teemu Palviainen, Noora Lindgren, Juha O. Rinne, Jaakko Kaprio

**Affiliations:** 1 Institute for Molecular Medicine Finland (FIMM), University of Helsinki, Helsinki, Finland; 2 Turku PET Centre, University of Turku, Turku, Finland; 3 Division of Clinical Neurosciences, Turku University Hospital, Turku, Finland; 4 Department of Public Health, Clinicum, University of Helsinki, Helsinki, Finland

## Abstract

This diagnostic study evaluates the association of reference panels with imputation quality for 2 single-nucleotide polymorphisms located on the apolipoprotein E (*APOE*) gene.

## Introduction

Given the importance of the apolipoprotein E (*APOE*) gene for risk of Alzheimer disease, determining this genotype is important in cognitive aging studies. Before the genome-wide genotyping era, the *APOE* gene (alleles ε2, ε3, ε4) was directly genotyped and defined by 2 single-nucleotide polymorphisms (SNPs), rs429358 and rs7412, in chromosome 19. Owing to rapid development of genotyping technology, the price of genome-wide arrays covering approximately 500 000 SNPs has decreased to less than $100, making it more cost-effective compared with direct genotyping of a single gene, such as *APOE*. In addition to genotyped SNPs, the information can be used to impute common nonmeasured variants such as rs429358 and rs7412 that are not directly genotyped on many chips. However, imputation accuracy depends on the genome-wide arrays, quality control, and reference samples.^1,2^

In this diagnostic study, we evaluated the association of reference panels with imputation quality of rs429358 and rs7412 by comparing imputation based on 3 different reference panels: 1000 Genomes (1000G),^3^ Haplotype Reference Consortium (HRC),^4^ and the Finnish-specific Sequencing Initiative Suomi (SISu).^5^

## Methods

We used a population-based older Finnish Twin Cohort^6^ study to examine the correspondence between rs429358 and rs7412 directly genotyped using a Sequenom (Taqman) and imputed rs429358 and rs7412 using 1000G Phase III version 5, HRC release 1.1, and SISu reference panels. Raw genotype data in a larger sample (5343 participants) using 5 array versions (12 v1.0 A, 12 v1.1 A, 24 v1.0 A, 24 v1.1 A, and 24 v1.2 A) of HumanCoreExome (Illumina) were merged before the quality control phase. We removed variants with call rate less than 97.5%, samples with call rate less than 95%, variants with minor allele frequency less than 1%, and variants with Hardy-Weinberg equilibrium *P* < 1.0 × 10^−6^. We removed samples with heterozygosity test method-of-moments *F* coefficient estimate values less than −0.03 or greater than 0.05, multidimensional scaling principal component analysis outliers, and samples that failed sex check. The number of genotyped autosomal variants after quality control was 239 894 (5328 participants).

We then performed prephasing using Eagle software version 2.3 (Broad Institute) and imputation with Minimac3 software version 2.0.1 (University of Michigan) (Table 1). The study sample (1704 participants) with directly genotyped rs429358 and rs7412 was extracted from each imputed data set. Ethical approval was obtained from the ethical committee of the Hospital District of Southwest Finland, and participants gave written informed consent.

**Table 1.** Cross Tabulation of Directly Genotyped and Imputed Single-Nucleotide Polymorphism of rs429358 and rs7412 for 1704 Individuals^a^

Imputation Based	Genotyped, No. (%)			Total, No.
**1000 Genomes**				
rs429358	TT	CT	CC	
TT	1152 (99.74)	3 (0.26)	0	1155
CT	0	503 (100)	0	503
CC	0	0	46 (100)	46
rs7412	CC	CT	TT	
CC	1505 (100)	0	0	1505
CT	2 (1.03)	192 (98.97)	0	194
TT	0	1 (20.0)	4 (80.0)	5
**Haplotype Reference Consortium**				
rs429358	TT	CT	CC	
TT	1153 (99.83)	2 (0.17)	0	1155
CT	0	503 (100)	0	503
CC	0	0	46 (100)	46
rs7412	CC	CT	TT	
CC	1505 (100)	0	0	1505
CT	0	194 (100)	0	194
TT	0	1 (20.0)	4 (80.0)	5
**Sequencing Initiative Suomi**				
rs429358	TT	CT	CC	
TT	1153 (99.83)	2 (0.17)	0	1155
CT	0	503 (100)	0	503
CC	0	0	46 (100)	46
rs7412	CC	CT	TT	
CC	1505 (100)	0	0	1505
CT	0	194 (100)	0	194
TT	0	1 (20.0)	4 (80.0)	5

^a^ Genotypes were imputed to 1000 Genomes Phase III version 5,^3^ Haplotype Reference Consortium release 1.1,^4^ and Sequencing Initiative Suomi Finnish-only reference panels.^5^ Imputation to 1000 Genomes and Haplotype Reference Consortium reference panels was done using the University of Michigan Imputation Server. The Sequencing Initiative Suomi reference panel consists of 16 962 023 variants from 3775 high-pass whole-genome (depth up to 30×) sequences.

## Results

Participants were of European ancestry (mean [SD] age, 74.2 [4.9] years; 775 [45%] women). For directly genotyped individuals, 984 (57.7%) had ε3/ε3 genotype, 521 (30.6%) had ε3/ε4 or ε4/ε4, 171 (10%) had ε2/ε2 or ε2/ε3, and 28 (1.6%) had ε2/ε4. Allele frequencies were 0.060 for ε2, 0.765 for ε3, and 0.175 for ε4.

Based on 1000G, 1701 individuals (99.82%) had correctly classified alleles of both rs429358 and rs7412 (Table 1). Results were similar for HRC and SISu: 1702 individuals (99.88%) and 1703 individuals (99.94%) had correctly classified rs429358 and rs7412, respectively (Table 1).

Using the HRC reference panel, 1702 individuals (99.88%) had correctly classified *APOE* genotype and ε4 carrier status (Table 2). Two (0.12%) of the ε4 noncarriers based on directly genotyped SNPs were incorrectly classified as ε4 carriers.

**Table 2.** Cross Tabulation of Directly Genotyped and Haplotype Reference Consortium Reference Panel Imputation–Based *APOE* Status in 1704 Individuals

*APOE* Status (% Individuals)	*APOE* Status Based on Imputed Single-Nucleotide Polymorphisms, No.						Total
	ε2/ε2	ε2/ε3	ε2/ε4	ε3/ε3	ε3/ε4	ε4/ε4	
ε2/ε2 (0.29%)	4	0	1	0	0	0	5
ε2/ε3 (9.74%)	0	166	0	0	0	0	166
ε2/ε4 (1.64%)	0	0	28	0	0	0	28
ε3/ε3 (57.75%)	0	0	0	983	1	0	984
ε3/ε4 (27.88%)	0	0	0	0	475	0	475
ε4/ε4 (2.70%)	0	0	0	0	0	46	46

Abbreviation: *APOE*, apolipoprotein E.

## Discussion

This study found that by using arrays described in the Methods section, imputation to all 3 reference panels, 1000G, HRC, and SISu, yielded high imputation accuracy of rs429358 and rs7412, 2 SNPs needed to determine polymorphic *APOE* ε alleles. The number of Finnish samples do vary in different reference panels: 99 in 1000G Phase III, approximately 1900 in the HRC, and 3800 in the Finnish-only SISu. Considering 1000G Phase III yielded improved accuracy compared with Phase I.^1,2^ Our results also suggest that determination of *APOE* can be reached equally well with most recent freely available cosmopolitan reference panels compared with a population-specific reference panel. Still, all Finnish samples and inclusion of only 1 brand of arrays were also limitations, and these results should be confirmed in people with different ancestry.
